# Supplementary material for: Preparation of a Dmap-Catalysis Lignin Epoxide and the Study of Its High Mechanical-Strength Epoxy Resins with High-Biomass Content
Source: Polymers (Basel). 2021 Feb 28;13(5):750. doi: 10.3390/polym13050750 (PMC7957740; doi:10.3390/polym13050750)
Supplement: Supplementary file 1 [file polymers-13-00750-s001.pdf]

polymers

Supplementary Material

Preparation of a Dmap-Catalysis Lignin Epoxide and the Study of Its High Mechanical-Strength Epoxy Resins with High-Biomass Content

Lingxia Song [1,2,3,4,5], Yeyun Meng [1,2], Peng Lv [1,2], Weiqu Liu [1,*] and Hao Pang [1,*]

[1] Guangzhou Institute of Chemistry, Chinese Academy of Sciences, Guangzhou 510650, China; jnssllxx@163.com (L.S.); mengyeyun15@mails.ucas.ac.cn (Y.M.); lvpengtust@163.com (P.L.)
[2] University of Chinese Academy of Sciences, Beijing 100049, China
[3] Guangdong Provincial Key Laboratory of Organic Polymer Materials for Electronics, Guangzhou 510650, China
[4] CAS Engineering Laboratory for Special Fine Chemicals, Guangzhou 510650, China
[5] CASH GCC(Nanxiong) Research Institute of New Materials Co., Ltd., Nanxiong 512400, China
* Correspondence: liuwq@gic.ac.cn (W.L.); panghao@gic.ac.cn (H.P.)

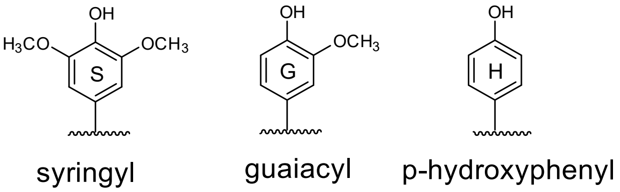

Figure S1. The typical structural units of lignin, and G, S and H represent guaiacyl, syringyl, and *p*-hydroxypheny units, respectively.

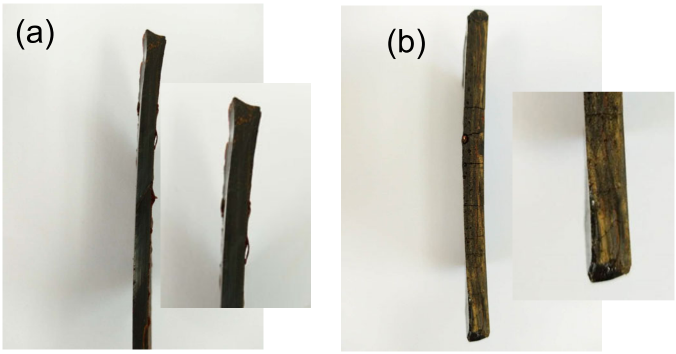

Figure S2. The structure morphology of both DMAP-lignin epoxide resin (**a**) and BTEAC-lignin epoxide resin (**b**). Compared to the BTEAC-lignin epoxide resin with many mud cracks and air holes, the DMAP-lignin epoxide resin has dense structure.

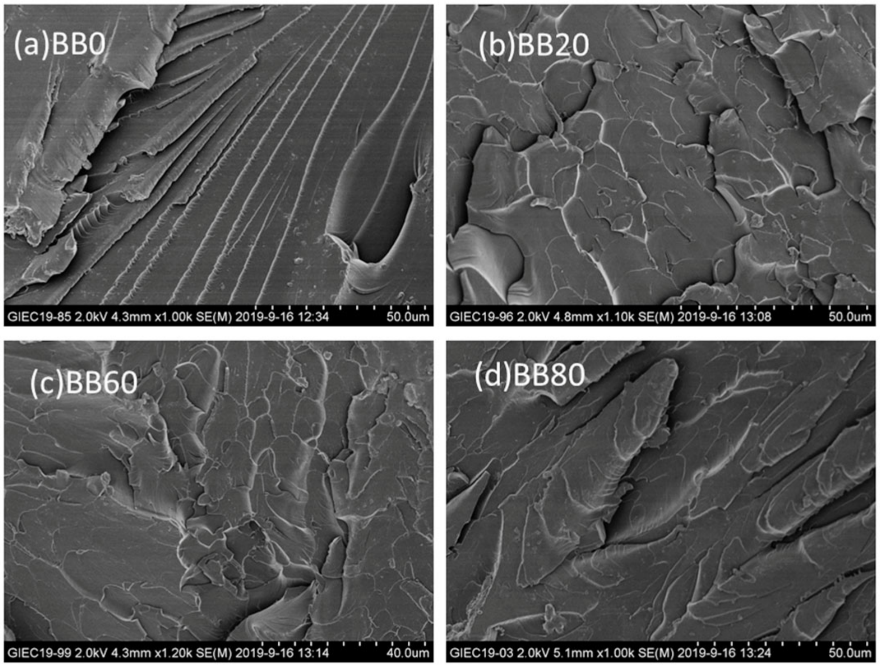

Figure S3. The SEM images of the fractured surface of the BB prepared with different addition amounts of BTEAC-lignin epoxide after tensile tests.
